# (Cost-)effectiveness of a combined lifestyle intervention integrated into standard care for low back pain compared to usual care for people with persistent non-specific low back pain who are overweight or obese: Protocol for a randomised controlled trial with parallel economic and process evaluations (Back2Health)

**DOI:** 10.1016/j.conctc.2026.101676

**Published:** 2026-08-01

**Authors:** Willeke Boonstra, Johanna M. van Dongen, Raymond W.J.G. Ostelo, Hidde P. van der Ploeg, Diederik H.R. Kempen, Femke van Nassau, Ivo J.Lutke Schipholt, Back2Health Consortium, Ömrüm Aydin, Maarten de Boer, Hana Broulikova, Pieter Coenen, Michiel W. Coppieters, Diyar Delawi, Barry van der Ende, Ruth Geuze, Harm Graat, Roel Hoogendoorn, Joep Kitzen, Mary Nicolaou, Karsten Ottink, Joost Rutges, J. Bart Staal, Pieter-Paul A. Vergroesen, Leen Voogt, Christopher Williams, Gwendolyne G.M. Scholten-Peeters

**Affiliations:** a Department of Human Movement Sciences, Faculty of Behavioural and Movement Sciences, Vrije Universiteit Amsterdam, Amsterdam Movement Sciences - Program Musculoskeletal Health, van der Boechorststraat 9, Amsterdam, 1081 BT, the Netherlands; b Department of Health Sciences, Amsterdam Movement Sciences, Faculty of Science, Vrije Universiteit Amsterdam, De Boelelaan 1085, Amsterdam, 1081 HV, the Netherlands; c Department of Epidemiology and Data Science, Amsterdam Movement Sciences, Amsterdam UMC, Vrije Universiteit Amsterdam, De Boelelaan 1117, Amsterdam, 1081 HV, the Netherlands; d Department of Public and Occupational Health, Amsterdam Public Health Research Institute, Amsterdam UMC, De Boelelaan 1117, Amsterdam, 1081 HV, the Netherlands; e Department of Orthopaedic Surgery, Joint Research, OLVG, Oosterpark 9, Amsterdam, 1091 AC, the Netherlands; f Department of Orthopaedic Surgery, Amsterdam University Medical Center, Amsterdam, the Netherlands

**Keywords:** Physiotherapy, Rehabilitation, Low back pain, Lifestyle, Randomised clinical trial

## Abstract

**Background:**

Low back pain is often influenced by unhealthy lifestyle factors, such as physical inactivity, poor sleep, unhealthy diet, and excess weight. Despite their impact, these factors are rarely addressed in routine care for non-specific low back pain. In the Netherlands, adults with overweight or obesity can access a combined lifestyle intervention targeting physical activity, diet, sleep and stress. Integrating this intervention into standard care for non-specific low back pain may improve patient outcomes.

**Methods:**

The Back2Health study is a two-armed randomised controlled trial with parallel economic and process evaluations evaluating the (cost-)effectiveness of an integrated lifestyle intervention versus usual care on physical functioning and physical activity over 36 months in patients with persistent non-specific low back pain and overweight or obesity. In total, 318 adults will be recruited from Dutch hospitals and randomised (1:1) to either the integrated lifestyle intervention, delivered by physiotherapists trained as lifestyle coaches, or usual care.

Co-primary outcomes are physical functioning and physical activity (steps/day) over 36 months. Secondary outcomes include pain intensity, systemic inflammation markers, sleep, and diet. Participants complete questionnaires every 3-6 months and attend in-person physical assessments at five timepoints. Data will be analysed according to both ‘intention-to-treat’ and 'per-protocol' principles. Additionally, a process and economic evaluation will be performed.

**Ethics and dissemination:**

The study protocol was approved by a medical ethics committee (METC Brabant: NL85373.028.23). Results will be published in peer-reviewed journals and presented at (inter)national conferences.

**Trial registration number:**

ClinicalTrials.gov (NCT06594796).

## 1 Background

Low back pain (LBP) represents a global healthcare challenge, being the predominant cause of years lived with disability worldwide. In 2021 alone, LBP was responsible for 70.2 million disability-adjusted life years worldwide [1]. The burden of LBP is exacerbated by various unhealthy lifestyle factors, including physical inactivity, stress, poor sleep, excess weight, and an unhealthy diet [[2], [3], [4], [5], [6], [7], [8], [9]].

International clinical guidelines recommend a conservative, non-pharmacological approach as first-line management for low back pain, emphasizing patient education, remaining active, and exercise within a biopsychosocial model of care [10]. Although lifestyle-related factors such as physical inactivity, obesity, smoking, sleep disturbances, and stress may contribute to the development and persistence of LBP, they are only briefly addressed due to limited evidence and implementation strategies. Similarly, in the Netherlands, LBP management focuses on primary care physiotherapy, exercise therapy, and general practitioner care according to guidelines to from the Royal Dutch Society of Physiotherapy (in Dutch: Koninklijk Nederlands Genootschap voor Fysiotherapie (KNGF)) [11] and the Dutch College of General Practitioners (in Dutch: Nederlands Huisartsen Genootschap (NHG)) [12]. While these guidelines encompass education, pain management, exercise therapy, and behavioural treatment, they do not address lifestyle-related issues in detail, due to limited evidence for these domains.

When primary care fails to relieve symptoms or an underlying pathology is suspected to cause LBP, patients are often referred to hospitals or rehabilitation centres for further evaluation and treatment. Approximately 65% of patients visiting hospitals in the Netherlands for LBP are overweight or obese [13]. These patients face increased disability, lower belief in the importance of physical activity, and heightened absenteeism, resulting in substantial societal costs [14,15]. This group of patients is often referred back to primary care, because medical specialists typically have limited treatment options to offer [16,17].

Recognizing the imperative to address both lifestyle and clinical factors becomes apparent in managing this subset of LBP patients. Such a combined approach may not only improve clinical outcomes, such as reducing pain and enhancing function, but can also offer broader health benefits by lowering the risk of diabetes and cardiovascular diseases [18,19]. This perspective aligns with recommendations from The Lancet series on LBP, which emphasize integrated, patient-tailored management strategies rather than isolated treatments [10].

Since 2019, overweight or obese adults in the Netherlands can follow a combined lifestyle intervention that incorporates guidance on physical activity, dietary habits, sleep, and stress [[20], [21], [22]]. In addition to individual- and group sessions, the intervention also incorporates evidence-based behavioural change techniques, such as eHealth self-monitoring, activity tracking, goal setting, action planning, and relapse prevention to increase effectiveness [[20], [21], [22], [23], [24]]. While conventional treatments such as exercise, education, and medication (i.e., usual care) have demonstrated effectiveness in managing persistent LBP [10,25], their effect sizes remain modest [26,27]. International studies have shown the potential of lifestyle interventions delivered by physiotherapists to be (cost)-effective compared to usual care, particularly in overweight or obese patients with persistent LBP [9,28]. However, these studies primarily evaluated short-term interventions, targeted only a limited number of lifestyle factors, and did not integrate the lifestyle intervention into routine guideline-based LBP care, limiting their applicability to everyday clinical practice.

This pragmatic study, referred to as Back2Health, will investigate the effectiveness of a combined lifestyle intervention integrated into standard care for non-specific LBP according to Dutch guidelines for patients with persistent non-specific LBP who are overweight or obese compared to usual care on self-reported physical functioning and physical activity levels over 36 months. The study specifically targets patients who are overweight or obese, as these individuals are currently eligible for a combined lifestyle intervention that is fully reimbursed through the Dutch basic health insurance package, providing an opportunity to integrate lifestyle supportwithin standard LBP care. Self-reported physical functioning and physical activity were selected as the primary outcomes because they align with the main aims of the Back2Health intervention: improving daily functioning and promoting a more active lifestyle. Physical activity is also considered a key mechanism for achieving long-term benefits. Additionally, cost-effectiveness of the intervention compared to usual care will be evaluated. A process evaluation will be conducted alongside the trial to study the implementation of the intervention into clinical practice.

## 2 Methods

### 2.1 Study design

The Back2Health study is a pragmatic multi-centre, two-arm, prospective randomised controlled trial with a superiority design. The Standard Protocol Items: Recommendations for Interventional Trials (SPIRIT) statement has been used for reporting [29]. The pragmatic design was chosen to evaluate the effectiveness, cost-effectiveness and implementation of the intervention under routine clinical conditions in the Netherlands, where eligible individuals with overweight or obesity can access fully reimbursed combined lifestyle interventions and choose between several nationally approved programmes [11,12,[20], [21], [22]]. Accordingly, flexibility in intervention delivery and co-interventions is preserved to reflect usual care and enhance the generalisability of the findings. The latter is particularly important when cost-effectiveness is also evaluated, because economic evaluations aim to inform real-world decision-making and therefore require estimates of costs and effects that are representative of routine clinical practice.

### 2.2 Participants

Patients will be recruited from secondary hospitals throughout the Netherlands from January 2025 till December 2026.

Overweight or obese patients with persistent (≥3 months) non-specific LBP, who experience decreased physical functioning, and are referred back from hospital care to primary care, are eligible for inclusion. Patients need to be at least 18 years of age and have either a body mass index (BMI) ≥30 (i.e., obese) or a BMI ≥25 (i.e., overweight) with at least one of the following factors: osteoarthritis, sleep apnea, risk factors for cardiovascular diseases or type 2 diabetes in line with the reimbursement criteria for combined lifestyle interventions in the Netherlands [30]. Non-specific low back pain was defined according to the Dutch general practice and physiotherapy guidelines as low back pain without a clearly identifiable specific anatomical or pathological cause explaining the symptoms [11,12]. Classification into specific or non-specific low back pain was made by the recruiting medical specialist based on clinical assessment, history, physical examination, and available diagnostic information in accordance with routine clinical practice. Additionally, patients need to experience an average LBP intensity of ≥3 out of 10 on a numeric pain rating scale (NPRS) over the past week and a physical functioning score of ≥4 out of 24 on the Roland-Morris Disability Questionnaire (RMDQ).

Patients will be excluded if they are diagnosed with specific LBP (e.g., fracture, radiculopathy, tumour, or infection), have a psychiatric condition that, in the clinical judgment of the recruiting medical specialist, is considered likely to interfere with study participation (e.g., schizophrenia), are currently pregnant, or are less than nine months postpartum. No predefined diagnostic criteria or questionnaire cut-off scores were used for this exclusion criterion.

### 2.3 Intervention

#### 2.3.1 Experimental group: Combined lifestyle intervention

The experimental group will follow one of five nationally-registered combined lifestyle interventions (i.e., BeweegKuur, Coaching op Leefstijl (CooL), SLIMMER, Samen Sportief in Beweging (SSIB) or X-Fittt), integrated into standard care for LBP, according to Dutch clinical guidelines for LBP [11,12,[20], [21], [22]]. The overall aim of the combined lifestyle intervention is to counsel participants who are obese or overweight to help them achieve a healthier lifestyle. For the purpose of this study, the intervention was delivered by physiotherapists who are additionally certified as lifestyle coaches through a accredited training programme. This training includes formal education, supervised practice, and competency assessment in behaviour change techniques. The intervention is based on the Dutch combined lifestyle intervention (GLI), in which motivational interviewing is the primary behaviour change technique, supplemented by goal setting, action planning, and problem solving. These techniques are applied as part of routine clinical practice by certified lifestyle coaches. The programs follow a personalised approach, guiding participants to explore their current lifestyle habits and how these affect their well-being, and set goals to adopt healthier behaviours. They are stimulated to take responsibility and remain in charge of their personal goals. The intervention spans two years. During the first six to twelve months, sessions focus on identifying unhealthy behaviours related to physical activity, diet, sleep, and stress, and on supporting participants in making sustainable lifestyle changes. The remaining months of the program emphasize goal monitoring and the maintenance of newly adopted lifestyle habits. The program consists of both individual and group sessions; however, the exact contact moments, frequency, and specific content or structure of the sessions will differ between the various combined lifestyle interventions.

In addition to the combined lifestyle interventions, the participants will receive standard care for LBP according to the national guidelines of the Royal Dutch Society of Physiotherapy [11] and the Dutch College of General Practitioners [12]. These guidelines recommend an active approach to treatment, including paineducation (i.e., education about low back pain, prognosis, and self-management) and exercise therapy [11,12]. It might also include behavioural interventions or non-exercise therapy interventions, depending on the individual treatment goals and dominant prognostic factors. Participants also maintain access to other usual care options during the study period, such as pain medication and hospital treatment.

#### 2.3.2 Control group: Usual care

The control group will receive care as recommended by the medical specialist, which may range from a watchful waiting approach to referral for physiotherapy, exercise therapy or general practitioner care, in accordance with current Dutch guidelines for LBP according to the Royal Dutch Society of Physiotherapy [11] and the Dutch College of General Practitioners [12]. It may also involve re-entry to the hospital after failed treatment in primary care. Similar to the intervention group, usual care follows a guideline-based, predominantly active approach that may include education and exercise therapy. However, it is delivered in routine clinical practice without the structured, multidisciplinary lifestyle coaching programme, predefined two-year follow-up structure, or systematic use of behaviour change techniques that are central to the intervention group.

### 2.4 Randomisation, blinding and treatment allocation

Participants will be randomised with a 1:1 ratio using a central computer-generated randomisation program by an independent researcher not involved in the study. A block randomisation approach (alternating block sizes of 6 and 4) will be used. Randomisation will be conducted after providing informed consent and completing the baseline measurement. Participants will be informed orally about their allocation. Treating medical specialists and general practitioners will be informed about the allocation of the participant. In this type of study, blinding patients and healthcare providers is not feasible; however, data analysis will be conducted by an investigator who is blinded to group assignments.

### 2.5 Data collection and outcome measures

Data will be collected at nine time points over the 36-month study period (i.e., 2 year intervention and 12 months follow-up), during which participants are asked to complete all questionnaires digitally via CastorEDC every 3 to 6 months (i.e. baseline and 3, 6, 9, 12, 18, 24, 30 and 36 months) (Castor Electronic Data Capture. https://castoredc.com). Physical measurements will be performed at five time-points (i.e. at baseline and 6, 12, 24, and 36 months) during visits either at the hospital or physiotherapist office, depending on planning and participant preferences. A description of the outcomes and time frame of data collection is provided in Table 1.

**Table 1:**
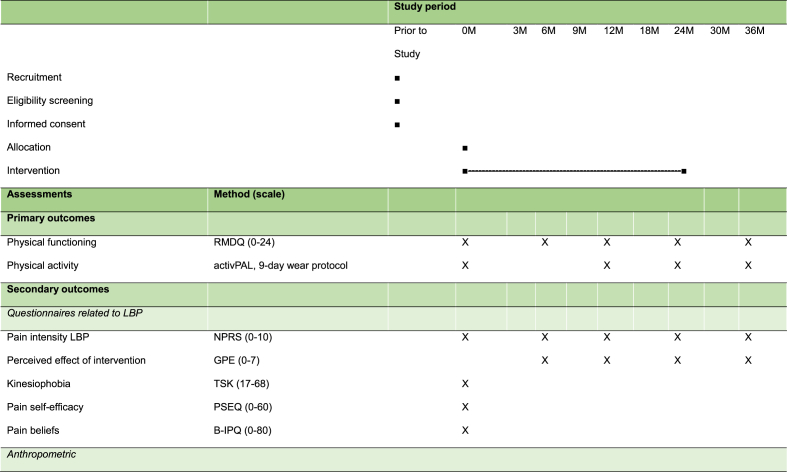

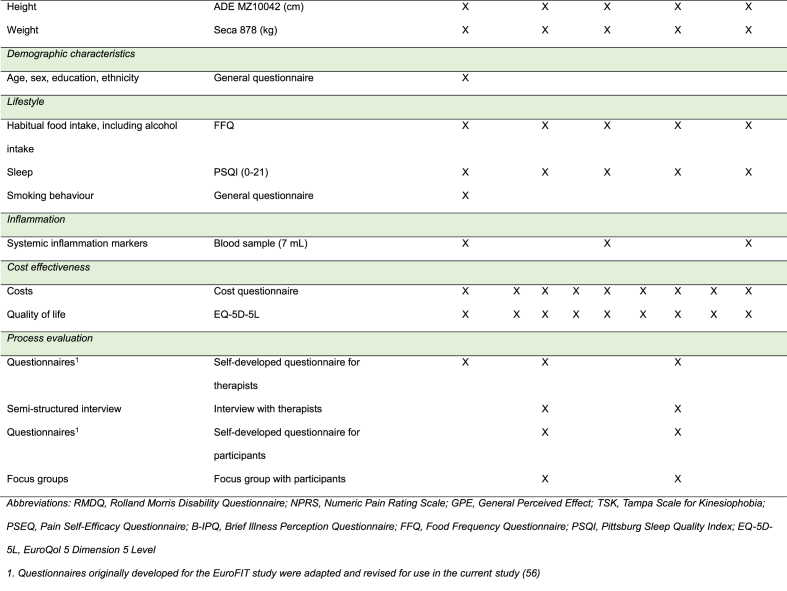
Summary of enrolment, intervention, and assessments, including all outcome measures [31].

#### 2.5.1 Co-primary outcomes: physical functioning and physical activity

The co-primary outcomes are physical functioning and physical activity (i.e., average steps per day over 7 days).

Physical functioning will be measured using the RMDQ [32]. This is the most important domain in the core outcome set for LBP [33]. The RMDQ is a 24-item self-report questionnaire about how LBP affects functional activities. Each question is worth one point so scores can range from 0 (no disability) to 24 (severe disability). The RMDQ is a reliable tool to measure the impact of LBP on physical functioning [34].

Physical activity will be assessed with the activPAL activity monitor (activPAL4, PAL Technologies LTD., Glasgow, U.K.) [35]. The activPAL measures physical activity and sedentary time through measuring accelerations and posture of the thigh [36]. The activPAL is a small non-invasive electronic logging device designed to quantify free-living daily activities. Participants follow a nine-day wear protocol, where the device will be worn consecutively, except during water-based activities (i.e., taking a bath, swimming). Excluding attachment and removal days, the device will be worn for 24 h for seven consecutive days at four time points (baseline and 12, 24 and 36 months).

#### 2.5.2 Secondary outcomes

Secondary outcomes will be assessed at the five main time points (baseline and 6, 12, 24 and 36 months) and include LBP intensity (NPRS), habitual food intake (FFQ), sleep habits (PSQI), quality of life (EQ5D5L), global perceived effect (GPE), systemic inflammation markers, and height and weight [[37], [38], [39], [40], [41]]. Pain beliefs (B-IPQ), pain self-efficacy (PSEQ) and fear of movement (TSK) will only be assessed at baseline [[42], [43], [44]]. An overview of all secondary outcomes is provided in Table 1.

#### 2.5.3 Cost-effectiveness

Cost-effectiveness of the intervention will be evaluated against usual care over 36 months. Costs will be measured from a societal and a healthcare perspective. From the societal perspective, costs will include the cost of the intervention, other healthcare use (i.e., primary, secondary, tertiary and medication), informal care, sports memberships and equipment, as well as the cost of productivity losses from paid (i.e., absenteeism and presenteeism) and unpaid work (e.g., voluntary work). From a healthcare perspective, only costs accruing to the formal Dutch healthcare sector will be included. Intervention costs will be estimated using micro-costing, which involves collecting detailed information on the quantity of resources used as part of the intervention (e.g., number of physiotherapy sessions) and their corresponding unit costs. Data on all other kinds of resource use will be obtained via iMTA-based cost questionnaires [45], tailored to the specific patient population and intervention. Resource use will be valued in accordance with the Dutch manual of Costing [46]. Quality-adjusted life years (QALYs) will be derived from the EQ-5D-5L. The EQ-5D-5L health states will be translated into utility values using the Dutch value set, with scores anchored at 0 (equivalent to death) to 1 (full health) [47]. QALYs will be computed by multiplying the duration spent in each health state by its associated utility value. Both questionnaires will be administered at baseline and at 3, 6, 9, 12, 18, 24, 30, and 36 months.

#### 2.5.4 Process evaluation

The process evaluation will study the implementation and mechanism of impact of the intervention, and will be carried out alongside the RCT with a mixed-methods approach using the UK MRC guidelines for process evaluation for complex interventions [48]. Both participants and physio therapists will be asked to complete questionnaires at baseline, 6 and 24 months. A selection of participants will be invited to join a focus group, and a selection of physio therapists will be invited for a semi structured interview about their experiences during the intervention, at 6 and 24 months. A trained independent researcher will conduct the focus groups and interviews. An overview of research aims and data collection is shown in Table 2.

**Table 2:**
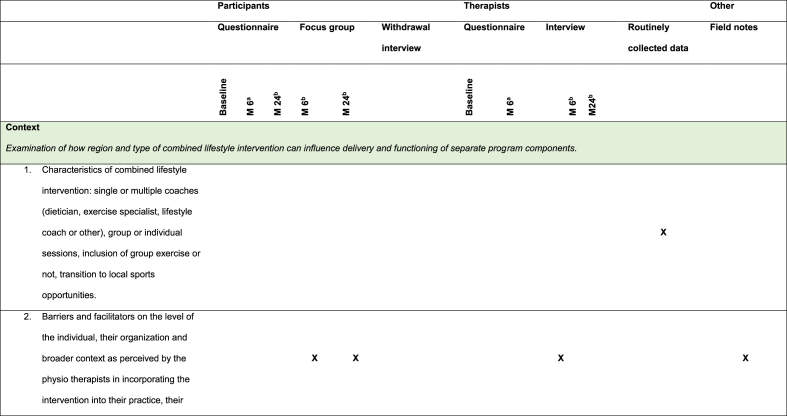

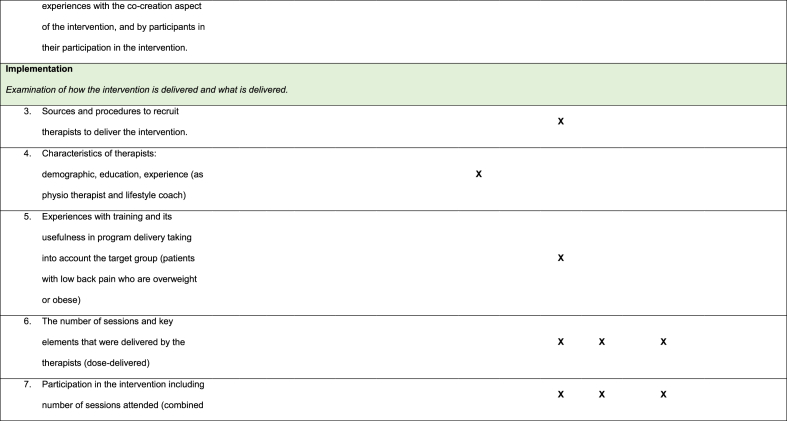

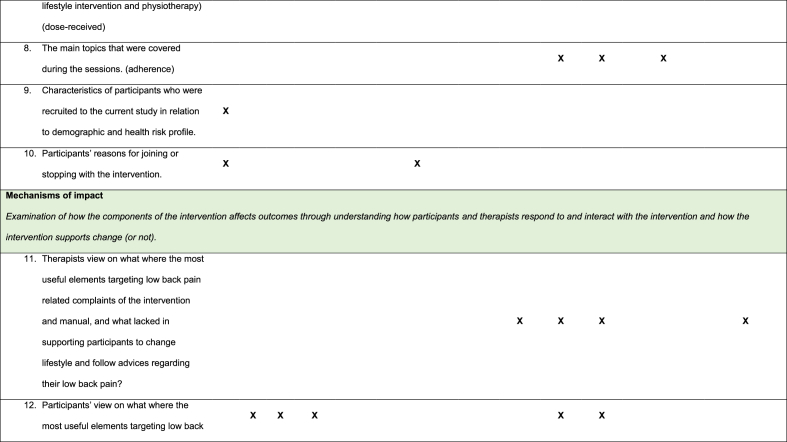

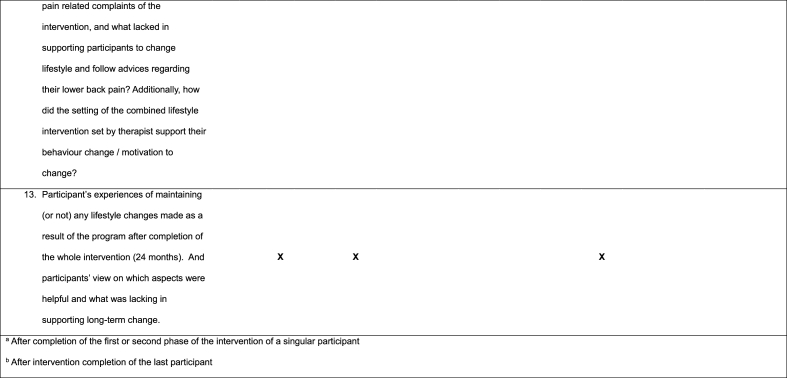
Research matrix and data collection methods for the parallel process evaluation.

### 2.6 Sample size calculation

To detect a between-group difference over a 36-month period of 3 points on the RMDQ (i.e., the smallest worthwhile between-group difference that would justify the implementation of the intervention), with a standard deviation of 6 [49], a power of 85%, an alpha of 2.5%, a crossover rate of 15%, and allowing for a drop-out of up to 25%, a total of 318 patients are needed (i.e., 159 in each group) [50]. To detect a between-group difference of 15% over 36 months for the other co-primary outcome (physical activity), i.e., average number of steps with a standard deviation of 2448 steps [51], a power of 85%, an alpha of 2.5%, cross-over rate of 15%, and allowing for a drop-out of up to 25%, a total of 302 patients are needed. Therefore, 318 patients will be recruited.

### 2.7 Statistical analysis

Data will be analysed in Rstudio. Primary analyses will be conducted according to the intention-to-treat principle and secondary analyses based on a per-protocol approach. Descriptive statistics will be used to describe baseline characteristics of the study population. Missing data for all outcomes and cost measures will be imputed using Multivariate Imputation by Chained Equations. All statistical tests will be two-tailed. Statistical significance for the co-primary outcomes is set at p < 0.025 to account for multiple testing.

#### 2.7.1 Primary and secondary outcomes

Linear mixed models with at least a two-level structure (i.e., time clustered within patients) will be used to analyse both co-primary outcomes; i.e., the difference in physical functioning and mean number of steps over a 7-day period during the 36-month treatment and follow-up period. The necessity of adding a third level (i.e., hospital) will be assessed using the −2 log-likelihood test. Time-by-group interaction terms will be used to detect differences between the groups at the various time points. Analyses will be adjusted for baseline confounders.

Secondary outcomes will be assessed using linear and logistic mixed models (depending on the nature of the dependent variable) using the same procedures as described above. Subgroup analyses will be performed to assess potential differences in outcome measures between people with different socio-economic status and the severity of LBP. Participants will be dichotomized into moderate or severe disability due to LBP based on a cut-off value of 40% on the RMDQ [52]. A RMDQ score of ≥40% (10 out of 24) indicates severe disability, while a score of <40% (9 out of 24) indicates moderate disability due to LBP. To test potential biases due to deviations from the intended intervention (e.g., crossovers), a per-protocol analysis will be performed in addition to the main intention-to-treat analysis.

#### 2.7.2 Cost-effectiveness

The economic evaluation will be conducted in accordance with the Dutch Guideline for Economic Evaluations [53] and using the R codes developed by Jornada Ben et al. (2023) [54]. Analyses will be conducted from a societal and healthcare perspective for both the co-primary outcomes (i.e., physical functioning and physical activity) and QALYs. Cost and effect differences will be estimated simultaneously using a bivariate model, while accounting for the skewed nature of costs (using non-parametric bootstrapping), missing data (using MICE), baseline imbalances (using regression-based adjustment), and the clustered nature of data (using mixed models), if necessary [55]. Then, incremental cost-effectiveness ratios (ICER) will be calculated by dividing the difference in costs by the difference in effects. The uncertainty around the ICER and 95% confidence intervals surrounding the cost differences will be estimated by bias-corrected and accelerated bootstrapping analysis with 5.000 replications and graphically presented on cost-effectiveness planes [55]. Cost-effectiveness acceptability curves will be constructed, to assess the probability that the combined lifestyle intervention is cost-effective compared to usual care at different values of willingness to pay.

#### 2.7.3 Process evaluation

The process evaluation follows a mixed method design [48]. Quantitative data obtained through the number of attended sessions, baseline and post-treatment questionnaires will be analysed using descriptive statistics.

The focus groups with participants and the semi-structured interviews with physio therapists will be analysed using thematic analysis [56]. First, concepts identified in the transcripts will be open coded. Similar concepts can receive the same code through axial coding. Thereafter, code patterns can be identified to create sub-themes, and will be discussed within the research group. Conceptually similar sub-themes can be grouped into overarching themes. Each theme will be analysed further and checked against all made codes and the dataset. Additional coding will be conducted if necessary. A second researcher will perform a concordance check on the final developed themes.

### 2.8 Monitoring

Installing a data monitoring committee was not warranted due to the conservative and non-pharmacological nature of the intervention. Data monitoring will be done by an independent researcher once a year. All (Serious) Adverse Events reported by the participant or observed by the researcher will be recorded and reported. Serious Adverse Events will be reported to the Medical Research Ethics Committee (in Dutch: METC).

### 2.9 Patient involvement

Patient representatives from the Dutch Association for Back Pain Patients (*Nederlandse Vereniging van Rugpatiënten ‘de Wervelkolom’*) and the Dutch Association for Overweight and Obesity (*Nederlandse Vereniging voor Overgewicht en Obesitas*) are members of the study consortium. They were actively involved in the design phase of the trial to help ensure that the intervention and research procedures are aligned with the needs and perspectives of the target population. Their involvement extends throughout the study, including advising on patient engagement strategies during recruitment, contributing to the development of accessible study materials, and supporting the dissemination of results to broader patient communities.

### 2.10 Data management

A data management plan has been developed to ensure that data handling aligns with FAIR (Findable, Accessible, Interoperable and Reusable) principles. At baseline, each participant will be assigned a unique, computer-generated identification code that bears no relation to personal data. All collected data will be pseudonymised using this code. Data include signed informed consent forms, questionnaires and physical measurement reports. These will be stored in CastorEDC, a secure data management system (Castor Electronic Data Capture. https://castoredc.com). Baseline and follow-up data will be entered into this system, and data obtained from the ActivPAL will be exported directly to a secure research drive accessible only to the research team at Vrije Universiteit Amsterdam. To ensure data quality, the online questionnaires include pre-programmed data constraints, and frequent consistency checks are performed.

### 2.11 Ethics and dissemination

The study protocol was approved by a medical ethics committee (METC Brabant: NL85373.028.23) and has been registered at ClinicalTrials.gov (NCT06594796). Important protocol modifications will be approved by the METC before implementation. All participants will provide informed consent before trial inclusion. Participants retain the right to request for deletion of their data. Study data will be archived for 15 years after study inclusion. Trial datasets will be made available upon reasonable request.

Results of the study will be published in peer-reviewed scientific journals and presented at (inter)national conferences. At the end of the study, participants will receive their own data regarding their health outcomes upon request.

## 3 Discussion

This paper describes the protocol of the Back2Health study, which is designed to investigate the (cost-)effectiveness of a combined lifestyle intervention integrated with standard care for LBP according to national guidelines for patients with persistent non-specific LBP who are overweight or obese and referred back from secondary care to primary care.

This is the first RCT to integrate a lifestyle intervention into clinical care for LBP. A systematic review including three RCTs evaluating lifestyle interventions for LBP showed only modest results regarding the clinical effectiveness of a lifestyle intervention in reducing LBP outcomes [28]. However, the interventions that were studied varied highly in dosage (i.e., number of sessions and duration of intervention), content (training program or telephone-based coaching), professionals involved (i.e,. mono or multidisciplinary) and country of implementation. In addition, these interventions were generally of shorter duration and focused on a limited number of lifestyle factors. In contrast, the current intervention evaluates a two-year multidisciplinary, personalised lifestyle programme integrated into standard LBP care, targeting multiple lifestyle domains (physical activity, diet, sleep, and stress) using behaviour change techniques."

Previous studies on the effectiveness of the combined lifestyle intervention have demonstrated its effectiveness in supporting adults in adopting more sustainable behaviours, leading to weight reduction and improvements in lifestyle factors, such as diet and physical activity [21,22]. However, while excess weight is a known prognostic factor for LBP, prior research also suggests that differences in physical functioning and physical activity levels significantly impact the quality of life in this patient population [3]. Therefore, both outcomes have been selected as co-primary outcomes to examine the effects of the intervention in overweight and obese LBP patients.

A major strength is that the intervention builds on the existing combined lifestyle interventions and other existing interventions recommended in national guidelines, which increases the chance of successful implementation when the intervention is deemed (cost-)effective. However, since the combined lifestyle intervention is not currently tailored to our patient group (i.e., people with persistent LBP), a process evaluation is needed to gain insight into the implementation and usefulness of its various components. In addition, the inclusion of multiple distinct combined lifestyle intervention programs to better reflect routine clinical practice may lead to variability within the experimental intervention arm, potentially introducing heterogeneity in the outcomes. Furthermore, the control arm receives usual care without restrictions, aside from being asked to not participate in combined lifestyle interventions. Consequently, control participants may still engage in lifestyle modifications independently, including consulting health professionals such as dieticians or personal trainers. This may attenuate the observed differences between the intervention and control arm. In response, healthcare usage (e.g., medical, allied health, medication and other) will be systematically recorded in both study arms.

A potential limitation could be that participants may perceive the 36-month duration of the trial as too long. To mitigate this, questionnaires and measurements have been carefully selected to minimize participant burden. Additionally, to maintain engagement throughout the study, participants will receive postcards on special occasions (e.g., birthdays and holidays) and periodic updates.

In summary, the Back2Health trial will provide much needed insight in the (cost-)effectiveness of a comprehensive, personalised treatment integrating lifestyle and clinical factors) for people with chronic non-specific LBP who are also overweight or obese, compared to a usual care approach. The trial will evaluate its impact on clinical outcomes, including self-reported physical functioning and objectively measured physical activity. Integrating lifestyle into the treatment of low back pain may also provide an opportunity to address lifestyle-related comorbidities that co-occur in this population, contributing to broader improvements in health and wellbeing.

## Funding statement

This work was supported by 10.13039/501100001826ZonMW grant number 10390022210014.

## CRediT authorship contribution statement

**Willeke Boonstra:** Methodology, Writing – original draft, Writing – review & editing. **Johanna M. van Dongen:** Conceptualization, Funding acquisition, Methodology, Writing – review & editing. **Raymond W.J.G. Ostelo:** Conceptualization, Funding acquisition, Methodology, Writing – review & editing. **Hidde P. van der Ploeg:** Conceptualization, Funding acquisition, Methodology, Writing – review & editing. **Diederik H.R. Kempen:** Conceptualization, Funding acquisition, Methodology, Writing – review & editing. **Femke van Nassau:** Methodology, Writing – review & editing. **Ivo J.Lutke Schipholt:** Methodology, Writing – review & editing. **Gwendolyne G.M. Scholten-Peeters:** Conceptualization, Funding acquisition, Methodology, Writing – review & editing.

## Declaration of competing interest

The authors declare that they have no known competing financial interests or personal relationships that could have appeared to influence the work reported in this paper.

## Data Availability

No data was used for the research described in the article.
